# The relevance of traditional knowledge systems for ethnopharmacological research: theoretical and methodological contributions

**DOI:** 10.1186/1746-4269-6-32

**Published:** 2010-11-17

**Authors:** Victoria Reyes-García

**Affiliations:** 1 ICREA and Institut de Ciència i Tecnologia Ambientals, Universitat Autònoma de Barcelona, 08193 Bellatera, Barcelona, Spain

## Abstract

**Background:**

Ethnopharmacology is at the intersection of the medical, natural, and social sciences. Despite its interdisciplinary nature, most ethnopharmacological research has been based on the combination of the chemical, biological, and pharmacological sciences. Far less attention has been given to the social sciences, including anthropology and the study of traditional knowledge systems.

**Methods:**

I reviewed the literature on traditional knowledge systems highlighting its potential theoretical and methodological contributions to ethnopharmacology.

**Results:**

I discuss three potential theoretical contributions of traditional knowledge systems to ethnopharmacological research. First, while many plants used in indigenous pharmacopoeias have active compounds, those compounds do not always act alone in indigenous healing systems. Research highlights the holistic nature of traditional knowledge systems and helps understand plant's efficacy in its cultural context. Second, research on traditional knowledge systems can improve our understanding of how ethnopharmacological knowledge is distributed in a society, and who benefits from it. Third, research on traditional knowledge systems can enhance the study of the social relations that enable the generation, maintenance, spread, and devolution of cultural traits and innovations, including ethnopharmacological knowledge.

At a methodological level, some ethnopharmacologists have used anthropological tools to understand the context of plant use and local meanings of health and disease.

I discuss two more potential methodological contributions of research on traditional knowledge systems to ethnopharmacological research. First, traditional knowledge systems research has developed methods that would help ethnopharmacologists understand how people classify illnesses and remedies, a fundamental aspect of folk medicinal plant selection criteria. Second, ethnopharmacologists could also borrow methods derived from cultural consensus theory to have a broader look at intracultural variation and at the analysis of transmission and loss of traditional ethnopharmacological knowledge.

**Conclusions:**

Ethical considerations in the ethnopharmacology of the 21st century should go beyond the recognition of the Intellectual Property Rights or the acquisition of research permits, to include considerations on the healthcare of the original holders of ethnopharmacological knowledge. Ethnopharmacology can do more than speed up to recover the traditional knowledge of indigenous peoples to make it available for the development of new drugs. Ethnopharmacologists can work with health care providers in the developing world for the local implementation of ethnopharmacological research results.

## Background

Ethnopharmacology is, by definition, at the intersection of the medical, natural, and social sciences [1]. Despite the interdisciplinary nature of ethnopharmacology, much of its research has been exclusively based on the combination of the chemical, biological, and pharmacological sciences. Less attention has been given to the potential contributions of the social sciences, including anthropology and the study of traditional knowledge systems (but see, for example, the work of Giovannini and Heinrich [2], Thomas, Vandebroek, and colleagues [3,4], Pieroni and colleagues [5], Albuquerque and Oliveira [6], Pardo-de-Santayana and colleagues [7] among others). When anthropological expertise and tools have been used, the main purpose has been to obtain catalogues of medicinal plant uses, which were often abstracted from their cultural contexts and subject to little analysis or interpretation [8-10]. Furthermore, more often than not -and especially when working among indigenous peoples- the sole purpose of obtaining those lists and catalogues has been to facilitate the intentional and focused discovery of active compounds. In sum, with some remarkable exceptions and without undervaluing researchers who have catalogued the often threatened knowledge of medicinal plant uses, to date many ethnopharmacologists have limited themselves to document indigenous pharmacopoeias in the search for pharmacologically unique principles that might result in the development of commercial drugs [11] or nutraceuticals [12].

Several reviews of the development of the discipline have warned against the disciplinarily bias in ethnopharmacology. For example, in a review of articles published in one of the flagship journals of the discipline, the *Journal of Ethnopharmacology*, Etkin and Elisabetsky [1] stated:

*Mission statement notwithstanding, during the first two decades of its existence most of the articles published in the JEP were not interdisciplinary. Two retrospective content analyses of the journal revealed for the periods 1979-1996 and 1996-2000 an increasing number of articles dedicated exclusively or primarily to pharmacology and pharmacognosy. More significant to the present discussion is the consistently small number of multi- or interdisciplinary articles, 4-6% of the total published *(pg 24).

Almost a decade later, the situation seem not to have changed much, as the editorial of a 2010 issue of the same journal [13] states that

*[Since its origins] numerous studies in the Journal dealing with medicinal and other useful plants as well as their bioactive compounds have used a multitude of concepts and methodologies. In many cases these were interdisciplinary or multidisciplinary studies combining such diverse fields as anthropology, pharmacology, pharmacognosy.... pharmaceutical biology, natural product chemistry, toxicology, clinical research, plant physiology and others (see Soejarto, D.D., 2001, Journal of Ethnopharmacology 74: iii). However, many studies still only pay lip service to such interdisciplinary research and there still remains an urgent need to further strengthen the contributions made by anthropology and other social and cultural sciences as well as to explore the political and social implication of our research*.

That ethnopharmacologists are growing aware of theoretical and methodological biases in the discipline is an important first step. Even more important is that the growing awareness on those biases has paralleled a more fundamental change in the goals of ethnopharmacology. Namely, the initial bias towards the chemical, biological, and pharmacological sciences closely related to the understanding that the overarching goal of ethnopharmacology is the search of biologically active compounds of plants, fungi, animals, and mineral substances used in traditional medicines. But, as this new field of research grows, ethnopharmacologists become more conscious that finding active compounds should only be one of the goals of the discipline. Many ethnopharmacologists have been -and still are- pushing for changes in how the goals of ethnopharmacology are conceptualized [14-18]. For instance, in a relatively recent article, Etkin and Elisabetsky argued that the discipline now "strives for a more holistic, theory-driven, and culture- and context sensitive study of the pharmacologic potential of (largely botanical) species used by indigenous peoples for medicine, food, and other purposes"[1]. But ethnopharmacology can not achieve these new goals without simultaneously adopting theoretical and methodological contributions from the social sciences. Here, I aim to contribute to that effort by reviewing the potential theoretical and methodological contributions to ethnopharmacological research of a branch of a social science discipline: research on traditional knowledge systems.

### Theoretical contributions of the study of traditional knowledge systems to ethnopharmacology

I use the terms traditional knowledge and traditional knowledge systems to refer to the knowledge of resource and ecosystem dynamics and associated management practices existing among people of communities that, on a daily basis and over long periods of time, interact for their benefit and livelihood with ecosystems [19,20]. The term does not merely refer to information about human uses of plants and animals [20]. Rather, it includes a system of classifications, a set of empirical observations about the local environment, and a system of resource use and management. It also includes believes in non-human beings (i.e., spirits, ancestors, ghosts, gods) and on how they relate to society. The study of TKS parallels ethnopharmacology in that both fields of research initially emphasized descriptive accounts, but they are now moving towards a more hypotheses-driven research. Here I will focus on three theoretical contributions from research on TKS, highlighting their relation to ethnopharmacological research.

#### TK as a holistic system of knowledge

The first theoretical contribution relates to the holistic nature of traditional knowledge systems. As mentioned, TK, rather than a compilation of information about plants and animals, is a way to understand the world, or what we understand as "culture". Anthropologists state that culture patterns human behavior and -through it- affects human health and well-being. In traditional societies, an essential function of culture has been to establish and transmit a body of knowledge, practices, and believes regarding the use of locally available natural resources to improve health and nutritional status. Quantitative research on the topic highlights the effects of locally developed traditional knowledge on adult and infant health and nutritional status. For example, in my collaborative research among the Tsimane', a hunter-horticulturalist society in the Bolivian Amazon, we have found that the level to which an individual shares the knowledge of the group is associated to own nutritional status [21] and offspring's health [22]. That is, people who share larger amounts of the traditional knowledge developed by the group display better health -measured through objective and subjective indicators - than people who do not share as much knowledge.

Ethnopharmacology can draw two important theoretical conclusions from those research findings. First, notice that those findings are based in a broad measure of traditional knowledge, not on the targeted study of a plant or a group of plants with active compounds. That is, we did not conduct a pharmacological study of local medicinal plants and then include those with active compounds in our questionnaire. Furthermore, our measure of TK was not limited to medicinal plants. Rather, our measure included questions on a wide range of useful plants (medicinal, but also edible, construction, dyes, and plants with other uses). We interpret the positive association between our broad measure of TK and objective and subjective indicators of health as indications that medicinal knowledge systems are not built of isolated pieces of information, but rather constitute a complex body of knowledge linked to a larger coherent ensemble. The implication is then that identifying active compounds in a plant might be of good use for the pharmacological industry, but it might be of limited use for knowledge holders, because it is possible that for a given medicine to be effective in the local context, it requires the accompanying practices and beliefs that provide the medicinal "meaning" to the plant (*sensu *Moerman, see bellow). The first point I want to stress here, then, is that, while it is evident that many plants used in indigenous pharmacopoeias do have active compounds, it is also likely that those active compounds do not act alone in indigenous healing systems, but they partially act because they have a shared medicinal cultural meaning [23]. And, as it has been highlighted by previous researchers [10,23], the efficacy of a medicinal plant should be measured in a culturally appropriated way, and the failure to consider the cultural context within which plants are used can result in misunderstandings of a plant's efficacy. So, it is the complex system, rather than the intake of particular plants with active compounds, that might shape the health and well-being of TK holders.

The second related lesson to be drawn from the example above relates to the indigenous understanding of health. Indigenous peoples have sophisticated ideas of health and well-being. As also recognized for the World Health Organization, for many indigenous peoples, health is not merely absence of disease [24]. Health is a state of spiritual, communal, and ecosystem equilibrium and wellbeing [25], which probably explains why traditional pharmacopeias include remedies both to cure physical ailments (whether caused by spiritual or magical beings, or by the physical world) and to improve one's well-being (i.e., to protect infants from witches or evil spirits or to enhance hunting abilities). Furthermore, among indigenous peoples, the choice of a medical treatment is often explained by this complex understanding of health and the perceived causes of illness. For example, the Tsimane' choice of medical treatment is often related to the perceived cause of the illness. Common illnesses, caused by the natural world, can be cured by medicinal plants or drugs, whereas illnesses caused by spiritual beings can only be cured by the intervention of a traditional healer [26]. When a person gets sick, she is often first treated as if she suffered from a common illness. Plants (or pharmaceutical) remedies are administered sequentially or simultaneously, often without consultation from any expert. If the condition persists, the Tsimane' start being suspicious that the illness is caused by witchcraft, in which case, they seek the help of a traditional healer. So, physical symptoms are only one of the clues to be used when selecting a treatment and the perceived (natural or spiritual) causes of the illness might be more relevant in the selection of the treatment. In that sense, as Moerman and Jonas have highlighted [23,27,28], even plants without active compounds can have healing effects, in the same way that placebo medicines have healing effects in Western culture. Plants and medicines might be effective, not because of their pharmacology, but because of the cultural "meaning" (*sensu *Moerman 2007) assigned to them. To put it in Moerman's [23] words:

*However, the effectiveness of these plants as medicines is not simply a consequence of their pharmacology; they are not pills disguised as herbs. Botanical medicinal effectiveness is inevitably some varying combination of pharmacology and meaning. Neglecting either aspect of this effectiveness is to provide only a partial, and thereby an erroneous, view of the subject *(pg. 459).

In sum, research on TKS and its relation to the health of indigenous people suggests that the medicinal uses of plants, animals, fungi, and minerals are better understood if studied as a domain of knowledge embedded in the large body of cultural knowledge, practices, and beliefs of a group. The focus on testing the active compounds of indigenous pharmacopoeias conveys the idea that local medicines become meaningful only when pharmacologically validated, and thus diminishes traditional knowledge systems and indigenous explanations of the world. Thus, an important task ahead for ethnopharmacology is to contextualize uses and cultural perceptions of plants as a way to acknowledge that the intangible attributes of a species may be as important criteria for inclusion in indigenous pharmacopeias as its tangible attributes.

#### The distribution of Traditional Knowledge

The second theoretical contribution from research on TK that can help in the ethnopharmacological enterprise relates to the distribution of knowledge within a group. Recently, Heinrich and colleagues [29] claimed that *"minimally, any [ethnopharmacological] field study should examine how plant knowledge is distributed in a society, and include some sort of consensus analysis to highlight the difference between common and specialist knowledge" *(pg. 9). The legitimate question is "why?"

From research initiated in the 1970s and continued to this day, we know that there are differences in the amount of cultural knowledge that individuals' hold [30-34]. For instance, in a study in the Brazilian Amazon, Wayland [35] shows that knowledge and use of medicinal plants is concentrated among women because of their role as managers of household health. Some other variables that have been shown to correlate with intra-cultural variation of TK include market integration [36,37], kinship affiliation [38], age [39], schooling [40], positions in a social network [41], and -of course- level of specialization on the domain of knowledge [42-45]. For example, in a now classic study in a Tarascan community in Mexico, Garro [42] found important differences in the level of medical knowledge of curers and laypeople. Overall curers and laypeople shared a single system of beliefs, however, curers showed higher agreement among themselves in expressing this system than non-curers.

The implications of intra-cultural differences on how laypeople and specialists understand the causes, symptoms, and treatments of illnesses have been addressed in medical [46], but not so much in ethnopharmacological research. Three decades ago, Kleinman and colleagues [46] suggested that the models of sickness held by laypersons and specialists may differ in terms of perceptions of what caused the ailment, why it started, when it did, what it did to the person, how severe it was, what were the treatment options, what results were expected from treatment, and what were the fears about the illness. They stressed the critical importance of understanding potential differences between laypersons and specialists for the successful resolution of health problems. As they argued, the different understanding of illness between patients and specialists may be at the root of medical problems, particularly because different understanding of illnesses might result in patient lack of adherence to medical regimens.

Folk healers (i.e., herbalists, curers, shamans, and the like) have been the typical focus of ethnopharmacological research. Ethnopharmacologists have focused on folk healers under the assumption that they concentrate most ethnopharmacological knowledge. However, specialists have often been studied in isolation, giving little attention to how specialists relate, interact, and contrast with non-specialists. But if -as we have learned from research on the distribution of TK- specialists and non-specialists do not necessarily share the same body of knowledge, nor the same understanding on how to cure diseases, then the focus on specialists knowledge necessarily biases the type of information being collected in ethnopharmacological studies. Furthermore, this focus on specialists limits the possibility of understanding how the patterned distribution of ethnopharmacological knowledge within a society affects the health of the group.

Thus, the patterned distribution of TK has two important implications for ethnopharmacological research. The first implication relates, of course, to the selection of informants. If TK is unequally distributed, the amount and quantity of information one can obtain clearly depends on how much and what type of knowledge is held by the selected informants. Researchers have highlighted differences between laypersons and specialists, but -as in other domains of traditional knowledge- most likely other patterned differences exist. For example, men can give different explanations to illnesses symptoms and treatments than women, or young people might use different treatments than elders. Thus, minimally understanding how knowledge is distributed in a community should be an important consideration in ethnopharmacological research, which so heavily relies on locally provided information.

The second implication of the patterned distribution of knowledge for ethnopharmacological research is more theoretical. If ethnopharmacological knowledge is unevenly distributed, and if this uneven distribution is patterned, then one should expect that people in certain characteristics should benefit more from the ethnopharmacological knowledge of the group than people without those characteristics. It also implies that similarities and differences in the belief systems of specialists and non-specialists are likely to affect how treatment alternatives are perceived and utilized. All important issues that ethnopharmacology could potentially address.

#### Transmission of Traditional Knowledge

A third theoretical contribution from research on TK to ethnopharmacological research relates to the study of the social relations that enable the generation, maintenance, spread, and devolution of cultural traits and innovations, including ethnopharmacological knowledge. Researchers have hypothesized that, unlike biological traits, largely transmitted by a vertical path through genes, cultural information can be transmitted through at least three distinct -but not mutually exclusive- paths: 1) from parent-to-child (vertical transmission), 2) between any two individuals of the same generation (horizontal transmission), and 3) from non-parental individuals of the parental generation to members of the filial generation (oblique transmission) [47]. Oblique transmission can take the form of (a) one-to-many, when one person (e.g., a teacher) transmits information to many people of a younger generation or (b) many-to-one, when the person learns from older adults other than the parents [47].

So the question is "how is ethnopharmacological knowledge transmitted?" Some anthropologists have stated that folk biological knowledge, including knowledge about what constitutes an illness and how to cure it, is mainly transmitted by parents to offspring [48,49]. For example, in a study of a rural population in Argentina, Lozada and colleagues [50] analyzed the transmission of knowledge of medicinal and edible plants and concluded that family members (especially mothers) were the most important source of medicinal knowledge. Other researchers have argued that parent-child transmission might not be the dominant mode of cultural learning, at least when a person's total lifespan is considered [51]. Quantitative studies on oblique transmission of ethnobotanical knowledge are scarce and focus on the transmission of knowledge from one-to-many. For example, Lozada and colleagues [50] found that experienced traditional healers outside the family are the second important source for the acquisition of knowledge of medicinal plants. Last, several authors have argued that there are also social and evolutionary reasons to expect intra-generational transmission of some types of cultural knowledge [52,53]. Observational studies suggest that, in some domains, children learn a considerable amount from age-peers [48,54]. For example, children regularly teach each other tasks and skills during the course of their daily play [48]. In a study in Mexico [54], Zarger showed that siblings pass along extensive information to one another about plants, including where to find them, their uses, or how to harvest or cultivate them. In my own fieldwork, I have often observed children using plants for medicinal purposes, both for themselves and for they playmates, which would suggest that children also pass to each other information on curative plants. Research also suggests that, later in life, young adults turn to age-peers rather than parents for information. Specifically in situations of cultural change, age-peers -not elders- are most likely to have tracked changes and should provide the best information to navigate in the new context; information that sometimes updates or replaces information previously acquired from parents [47,51]. In sum, although previous empirical research has outlined the importance of the vertical path in the transmission of TK, theoretical models and empirical evidence from fields other than anthropology suggest that the importance of vertical transmission may be overstated [51], and that neither vertical nor oblique transmission should be expected to dominate across all domains [55,56].

The studies cited here also highlight that the selection of one type of transmission over another might depend both on the cultural group and the domain of knowledge examined. For example, medicines to cure illnesses from the natural world might be transmitted by a different channel than medicines to cure illnesses caused by spirits. Understanding the strategy selected by a society for the transmission of ethnopharmacological knowledge is important because each of those transmission pathways -or the way they are combined- affect differently the distribution, spread, and therefore maintenance of knowledge. For example, as is the case for other cultural traits [47], ethnopharmacological information vertically transmitted (i.e., from the parent to the child, or from one selected adult in the parent generation to one selected young, as many iniciatic systems) would be highly conservative. That is, because it is less shared, information vertically transmitted may maintain individual variation across generations. Furthermore, innovations and new information would experience slower rates of diffusion in a population when compared with horizontal or oblique transmission. By contrast, horizontal transmission might lead to fast diffusion of new information or innovations if contact with transmitters is frequent. Furthermore, vertical transmission is based in two models, whereas oblique and horizontal transmissions are based on larger samples, and larger samples might provide more accurate (less biased) information [57]. The combination of horizontal and oblique transmission involving many transmitters to one receiver would generate the highest uniformity in ethnopharmacological knowledge within a social group, while allowing for generational cultural change.

It is also possible that the strategies to transmit TK change over time. Theoretical modeling suggests that changing social contexts, as the ones that experience many indigenous societies nowadays with globalization and market integration, favor reliance on oblique rather than on vertical transmission [55]. For example, with increasing exposure to market economy and commercial drugs, ethnopharmacological knowledge might need to be used in new situations or in interaction with new products. To navigate cultural shifts, individuals might opt to select information that has been effective from a wider subset of the population (like non-parental adults). This shift might help ethnopharmacologists understand why indigenous pharmacopoeias heavily reliant on vertical transmission are threatened by modernization in a much deeper way that indigenous pharmacopoeias that have traditionally been transmitted through other pathways.

Last, research on the transmission of TK can also help ethnopharmacologists understand the different paths through which different types of knowledge are transmitted. For example, research among the Tsimane' suggests that ethnobotanical knowledge (such as names or traits used for plant recognition) and skills (or how to put this knowledge into practice) are not transmitted through the same paths [56]. Ethnobotanical knowledge might be easier to acquire than ethnobotanical skills and is mainly acquired during childhood. The acquisition of knowledge relies on cumulative memory and individuals can learn quickly and effectively through relatively few interactions; therefore, individuals can acquire ethnobotanical knowledge from many sources. The acquisition of skills might require higher investments by the learner. Acquiring skills is more costly in time and might require a number of direct observations and repetition within a particular context. So, individuals might be more conservative in selecting models for the transmission of skills and place more weight on information acquired from older or more experienced informants.

To sum up, a focus on understanding how ethnopharmacological knowledge is transmitted would open new research possibilities in ethnopharmacology. Specifically, quantitative data on the mechanisms of transmission of cultural traits could be useful in predicting within-group variability and stability of traditional pharmacopeias over time and space.

I now move to discuss how methodological contributions in the study of TKS can help in ethnopharmacological research.

### Methodological contributions of the study of traditional knowledge systems to ethnopharmacology

Ethnopharmacology has drawn on many tools from anthropology. The broad contributions of anthropology to ethnopharmacological research have been the subject of previous reviews [58] and critical assessments [59]. So here I would just make a general consideration on those tools, referring the reader to previous work for detailed information.

Previous researchers with anthropological training have argued that anthropology can make a unique contribution to ethnopharmacological research by providing the conceptual and practical tools that would allow ethnopharmacologists to develop the ethnography of plant use and of health and disease in sufficient depth to correlate with laboratory investigations of plant constituents and activities [58]. Among the many tools that anthropology can -and has- contributed to ethnopharmacology, researchers have highlighted that detailed ethnographic research is crucial in understanding traditional medical practices. As argued before, traditional medical systems are holistic in nature and often consider illness, healing, and human physiology as a series of interrelationships among nature, spirits, society, and the individual [60,61]. As Elisabetsky argued [62]

*Traditional remedies, although based on natural products, are not found in "nature" as such; they are products of human knowledge. To transform a plant into a medicine, one has to know the correct species, its location, the proper time of collection [...], the part to be used, how to prepare it [...], the solvent to be used [...], the way to prepare it [...], and, finally, posology [...]. Needless to say, curers have to diagnose and select the right medicine for the right patients *(pg. 10).

Ethnographic research -based on extensive field studies- has proven key to understand those relations and to assess how local people perceive, understand, classify, and use resources in their environments. Specifically, some of the qualitative and ethnographic methods more commonly used in ethnopharmacological research include participant observation, interviews with key informants, focus groups, structured and unstructured interviews, survey instruments and questionnaires, lexical and semantic studies, and discourse and content analysis (see [58,59,63,64].

In sum, although still underused [14], some of the anthropological tools that ethnopharmacologists can add to their toolkit to reveal the cultural construction of health and healing in diverse cultures have been already discussed by other researches. I would like to move now to discuss two methods frequently used in research on TKS whose contributions to ethnopharmacological research are not so commonly known: 1) folk classification and 2) cultural consensus analysis.

#### Ethnoclassification

In its broadest sense, ethnoclassification, or folk taxonomy, refers to how traditional communities identify, classify, categorize, and name the world around them. Ethnobiologists place folk taxonomies within the broader analysis of TK because folk taxonomies are considered to be reflections of how people organize their knowledge of the universe [32,65-68], and have large impacts on people's perceptions and actual behaviors [66]. Food taboos, for example, reflect local knowledge and perceptions of edible and inedible foods, which in turn impact subsistence, technology, the construction of social landscapes, social interactions, notions of prestige, and gender distinctions, among other behaviors [69]. Consequently, studies on folk taxonomy can provide insights into ethnopharmacology because folk taxonomy not only organizes and condenses information about the natural world, but it also provides a powerful systematic tool to examine the distribution of biological and ecological properties of organisms [66].

Studies on ethnoclassification have mostly documented how different cultural groups classify the environment, especially plants and animals. A seminal work on the topic is the research by Berlin, Breedlove, and Raven in the 1970s [67,70]. Based on ethnobotanical studies in Central and South America, those authors elaborated general principles of folk taxonomy and drew convincing parallels with Linnaean taxonomy. According to Berlin [71], humans respond to plant and animal diversity in their environment by grouping living organisms 1) into named categories that express differences and similarities between them and 2) into hierarchical classificatory categories of greater or lesser inclusion. Because native taxonomies differentiate taxa by broad morphological traits, there is often a strong correspondence between Linnaean and other folk taxonomies at the "generic-species" level [66,71]. Thus, folk classificatory systems retain a vast store of information about biology, ecology, and ethology of animals and plants. Berlin's principles, though not without critics, have been tested by other authors (e.g., [30,72]), and many studies throughout the world suggest that the folk classification of animals and plants are not arbitrary, but determined by some degree of biological reality or universal cognition.

But people do not only organize plants and animals into categories. One area where ethnoclassification can inform ethnopharmacological research relates to the classification of illnesses and medicines, and how this classification affects the selection of curative and preventive substances [9,10,73]. I will illustrate the point of how ethnoclassification can contribute to ethnopharmacological research through the example of the hot-cold humoral system.

Humoral folk medicinal models rest on the idea that illnesses are a consequence of some imbalance of intangible qualities of the body (or humors). Under this classificatory system, illnesses should be treated (or prevented) with medicines with opposite qualities [34,74]. For example, under the hot-cold system, a humoral folk medicinal model common in areas as diverse as Latin America [34] or China [74], health is believed to be a balance between hot and cold elements in the body, and illnesses appear when the body is too "hot" or too "cold." If the body is too "hot", balance can be restored by treatment with "cold" foods, remedies, or medicines, and viceversa. Under this humoral system, then, medicines are selected, not exclusively by their particular active properties, but also depending on where they fit in people's classification system.

Thus, understanding how people classify illnesses and remedies on humoral systems is key in ethnopharmacological research because those classifications are a fundamental -although not exclusive- part of medicinal plant folk selection criteria. For example, Ankli and colleagues [75,76] investigated hot/cold classifications and taste and smell perceptions of Yucatec Maya medicinal and non-medicinal plants. Their results show that non-medicinal plants were more often reported to have no smell or taste than medicinal plants: good odor was a sign of medicinal use and a large percentage of medicinal plants were reported to be astringent or sweet. Non-medicinal plants were rarely classified humorally and medicinal plants humoral qualities appeared to refer to a plant's classification. Ankli and colleagues found correlations between Mayan perceptions of taste and smell and known chemical constituents [75,77], but no specific group(s) of compounds was associated with alleged hot or cold properties of plants. Ankli and her colleagues concluded that taste and smell are important selection criteria for medicinal plants among the Maya, but they are not a central unifying principle of Maya medicinal plant classification. Shephard [78] has also documented the role of the senses in medicinal plant selection.

In sum, it is evident that there are often biological bases for medicinal plant selection, but folk classification also constitutes a fundamental part of medicinal plant folk selection criteria. A bigger emphasis in ethnoclassification would help ethnopharmacology to move from a narrow focus on "what plants are included in indigenous pharmacopeias?" to broader questions such as "why are those plants selected and used?"

#### Cultural Consensus Analysis

The second set of methods commonly used in research on TKS that offers interesting possibilities in ethnopharmacological research are methods derived from cultural consensus theory [79]. Cultural consensus theory was developed by anthropologists trying to estimate culturally correct answers for different domains of local knowledge [80]. The cultural consensus theory rests on several assumptions. First, there is a culturally correct answer for every question. Whatever the cultural reality is, it is the same for all informants and is defined as the answer given by most people [81]. Second, knowledge consists of agreement between informants. The level of agreement between informants reflects their joint agreement [38,82]. Third, the probability that an informant will answer a given question correctly is a result of that informant's competence in that domain of knowledge. Competence refers to the share of correct answers by the informant.

Information for the cultural consensus model consists of responses by informants to multiple-choice questions. A computer software, ANTHROPAC [83], calculates each informant's competence and establishes whether the domain of knowledge being analyzed is consensual. The cultural consensus model has been largely used in TKS research (see [84] for a review) and has also been used to analyze folk medical beliefs [44,85-88] and humoral classifications of illness [34]. However, and despite the importance that consensual responses have in ethnopharmacological research [23,89,90], cultural consensus analysis is still not widely used in ethnopharmacology.

Cultural consensus analysis would allow ethnopharmacologists a broader look at intracultural variation and at the analysis of transmission and loss of traditional ethnopharmacological knowledge. Cultural consensus analysis differs from other ways of examining consensual responses in a group in that it reflects the patterning of responses and variation around the cultural norm. Under the traditional knowledge-testing approach, informant's knowledge is described in terms of deviance from the biomedical model, but it does not allow distinguishing between errors that are due to a lack of biomedical knowledge and those that are due to different explanatory models. In contrast, cultural consensus analysis can identify items that are part of a group's explanatory model. In that sense, cultural consensus analysis could complete the traditional knowledge-testing approach. The traditional knowledge-testing approach allows researchers to assess individual performance in terms of biomedically correct answers; the cultural consensus analysis allows researchers to identify items that are part of a group's explanatory model.

## Conclusions

In this article I have tried to highlight theoretical and methodological, actual and potential, contributions of research on TKS to ethnopharmacological research. Let me now orient this last part to discuss the future of the discipline through the lenses of an anthropologist who specializes in the study of TK.

In commenting on a previous version of this paper, Moerman, Pieroni, and McClatchey highlighted to me the fact that there has not been a drug added to the Northern pharmacopoeia by any ethnobotanical or ethnopharmacological lead in probably half a century (Moerman, *comm. pers*., [91]) Furthermore, despite much ethnopharmacological research conducting bioevaluation of traditional drugs, traditional medicines and herbal drugs available on global and local markets are not -in large parts- isolated molecules resulting from bioevaluation, but rather raw dried herbs and plant-based extracts and fractions (Pieroni, *comm. pers*.) Yet the romance of ethnopharmacology as a pathway to develop new drugs out of the evaluation of traditional remedies persists in the minds of many. And one can not help but wonder whether this romance is just an attempt to justify the existence of a discipline that failed to meet its original goals.

Through the lenses of an anthropologist, that is, through the lenses of someone who is not necessarily interested in the bioevaluation of traditional medicines, there are -however- other possible futures for ethnopharmacology. In this article I have tried to discuss several research venues where ethnopharmacologists could contribute to improve our understanding cultural differences in perceptions, uses, and management of traditional remedies. Let me conclude by emphasizing the public health application that derives from the research suggestions made here.

While indigenous pharmacopoeias have historically contributed to the development of allopathic and herbal drugs thus adding to improve health in the global north, rarely ethnopharmacological expertise and findings are used to improve the long-run health in the regions of study. The consequence is that nowadays indigenous peoples suffer from the worst health status around the word [92-97].

Ethnopharmacologists have been fundamental in the widespread awareness of the ethical issues associated with documenting indigenous pharmacopoeias. Ethnopharmacologists and anthropologists have been among the first ones raising concerns about the compensation to indigenous people for the commercial uses of their traditional knowledge by pharmaceutical industries, about the need to develop appropriated mechanisms for the protection of indigenous people's intellectual property, and about the importance of conducting research in an ethical way (including issues such as asking for Prior Informed Consent and other relevant research permits granted by universities and governmental organizations [11,16,98-103]). That is, ethnopharmacologists, with ethnobiologists, have raised their hands against the commodification of the sacred, to use Posey's words [20]. As a response, international legal frameworks, such as the one established by the Convention of Biological Diversity, have been developed to safeguard the intellectual property of cultures and individuals with specialist knowledge.

As the discipline considers expanding its objectives from the intentional search of biologically active compounds of substances used in the traditional medicines to a more holistic and culture-sensitive study of the pharmacologic potential of those substances, ethnopharmacology should also incorporate new ethical considerations related to the new knowledge developed. Those considerations should go beyond the recognition of the Intellectual Property Rights of indigenous peoples or the acquisition of appropriated research permits, to include the healthcare of the original holders of ethnopharmacological knowledge. Many authors have highlighted the importance of culturally appropriate health services for indigenous peoples. In some regions of the world including Australia, New Zealand, Canada, Colombia, Ecuador, and Peru, new medical services are being implemented where indigenous medicine is practiced alongside allopathic medicine [93,95]. Ethnopharmacologists can be instrumental in working with health care providers in the developing world for practical implementation of ethnopharmacological research results.

In sum, ethnopharmacology can do more than speed up to recover the traditional knowledge of indigenous peoples to try to make it available for the development of new drugs in the North. Ethnopharmacology has the potential to contribute to the improvement of the health of indigenous peoples.

Let me finish quoting the words of Nina Etkin [14], as a tribute to someone who not only did invaluable, theoretical, methodological, and ethical contributions to the discipline, but also as a tribute to someone who was an inspiration to make ethnopharmacology more meaningful for local populations.

*Today, the interest that many pharmaceutical companies have in primarily developing-world diseases has more to do with implications for Western travelers than with indigenous populations who cannot afford expensive prophylaxis and therapy. Ethnopharmacologists could accept a challenge to turn this around. It would be provident at this juncture to address how the results of sophisticated medical ethnography and rigorous bioassays can be meaningfully integrated, translated, and applied to the traditional populations who use those plants *(pg. 182).

This should be, in my opinion, a primary goal of the discipline.

## Competing interests

The author declares that they have no competing interests.
